# An Italian Delphi consensus on the epidemiology, characteristics, and management of C3 glomerulopathy

**DOI:** 10.3389/fimmu.2026.1842797

**Published:** 2026-10-19

**Authors:** Luigi Biancone, Carlotta Galeone, Paolo Mariani, Claudio Pelucchi, Giuseppe Castellano

**Affiliations:** 1 Division of Nephrology Dialysis and Transplantation, Department of Medical Sciences, Città Della Salute e Della Scienza Hospital and University of Turin, Turin, Italy; 2 Bicocca Applied Statistics Center (B-ASC), University of Milano-Bicocca, Milan, Italy; 3 Department of Clinical Sciences and Community Health, University of Milan, Milan, Italy; 4 Department of Nephrology, Dialysis and Renal Transplantation, Fondazione IRCCS Ca’ Granda Ospedale Maggiore Policlinico, Milan, Italy

**Keywords:** C3 glomerulopathy, Delphi consensus, disease outcome, incidence, prevalence, rare diseases, unmet needs

## Abstract

**Background:**

C3 glomerulopathy (C3G) is a chronic kidney disease previously included within the broader spectrum of membranoproliferative glomerulonephritis. Information on various aspects of C3G, including epidemiology, management, and patient needs, is extremely scarce at the international and particularly at a national level.

**Methods:**

Between April 2024 and February 2025, we conducted an online iterative modified Delphi study involving Italian nephrologists with expertise in C3G. The Delphi study was performed according to the best practices for defining consensus and was reported following the ACcurate COnsensus Reporting Document (ACCORD) guidelines. Five main areas of investigation of C3G were explored, i.e., epidemiology, patient characteristics, disease outcomes, treatment, and other mixed aspects.

**Results:**

Of the 23 invited clinicians, 2 (8.7%) did not reply and 4 (17.4%) did not satisfy the inclusion criteria defined by the Steering Committee. Thus, the voting panel included 17 Italian experts. Two iterations were required to complete the Delphi study. All 17 panellists participating to the first iteration completed the second Delphi round. Agreement was achieved on an estimated number of 60–90 incident patient per year (94% agreement) and about 2700 prevalent cases of C3G (82% agreement) in Italy. The stabilization of eGFR and an improvement in proteinuria emerged as key disease and treatment outcomes. All experts agreed that the availability of specific and effective treatments and rapid and correct disease diagnosis are currently the most important unmet needs related to C3G.

**Conclusions:**

This investigation provided relevant information for improving our knowledge of C3G, stimulate discussion and enhance research on this ultra-rare disease in Italy.

## 1 Introduction

C3 glomerulopathy (C3G) is a rare kidney disease caused by dysregulation of the alternative complement pathway, leading to damage primarily confined to the glomeruli. It is associated with autoantibodies (nephritic factors) and genetic variants that stabilize the C3 convertase, promoting sustained complement activation (1, 2). C3G was classified about a decade ago (3, 4), and it was previously included within the broader spectrum of membranoproliferative glomerulonephritis (MPGN). It can be further divided into two main subtypes called C3 glomerulonephritis (C3GN) and dense deposit disease (DDD), which can be classified through electron microscopy (5).

C3G is an ultra-rare disease, with an estimated incidence around 1-1.5 cases per million person-years in Europe (6, 7). In consideration of its rarity and recentness of definition, available information on various disease aspects, including epidemiology, management, and patient needs, is extremely scarce, at an international and particularly national level. For example, to our knowledge, no data on the prevalence and/or incidence of C3G have been published to date in Italy.

Similarly, while estimated glomerular filtration rate (eGFR) slope and changes in proteinuria levels are recognized factors in the progression of several chronic renal diseases, thus representing relevant surrogate endpoints for kidney failure (8, 9), data supporting their use in the C3G setting are still scanty (10). In such circumstances, expert opinions obtained through rigorous methodologies, such as Delphi consensus, a renowned, iterative technique used in biomedical research and other fields (11), may play a supportive role to enhance disease understanding and management (12, 13).

Therefore, we conducted an online iterative modified Delphi study involving Italian nephrologists with expertise in C3G, with the aim to achieve consensus on several undefined or controversial topics related to the disease, with specific focus on—but not limited to—the national setting.

## 2 Methods

The Delphi study was performed according to the guidelines and best practices for defining consensus, considering anonymity of participants, multiple iteration, controlled feedback, and statistical stability of consensus, and the whole investigation was reported following the ACcurate COnsensus Reporting Document (ACCORD) guidelines (12, 14, 15). The protocol of the Delphi consensus was not registered in advance in any registration platform. Clinical trial number: not applicable.

The study was conducted in Italy and involved experts from several geographical areas throughout the Country, between April 2024 and February 2025. We hereby describe the phases and methods of the investigation, in line with the ACCORD scheme. A flowchart synthesizing each subsequent step of the Delphi process is also given in **Figure 1**.

**Figure 1:**
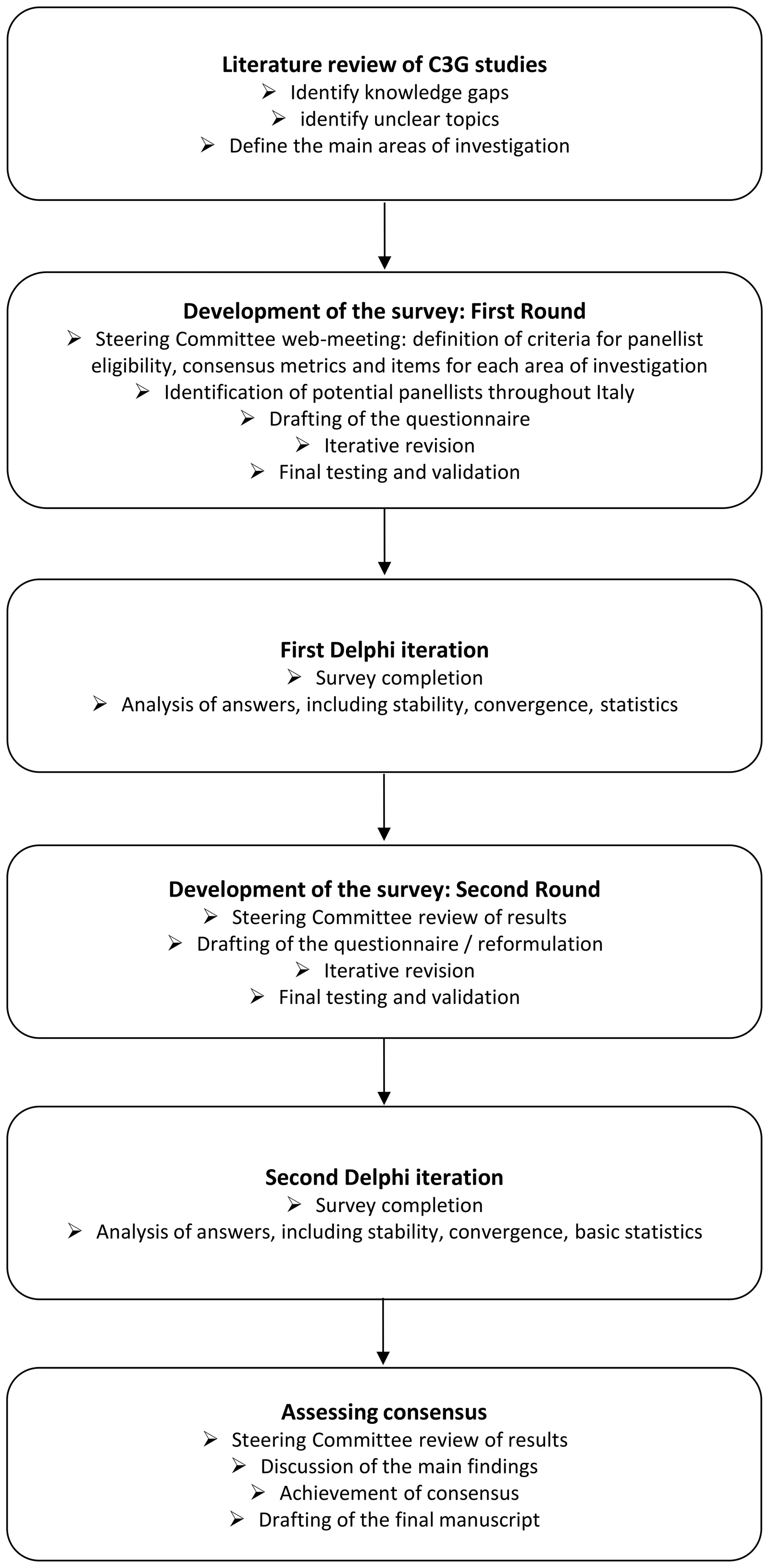
Flow-chart scheme outlining the subsequent steps of the Delphi process.

### 2.1 Preparatory research

A literature review of C3G studies, preliminary to the Delphi survey, was conducted between April and July 2024, with the aim to identify knowledge gaps and unclear topics related to the disease that warranted consideration in the consensus study. The search strategies were separated according to the different areas of investigation. A systematic review of the epidemiology of C3G in Western and Central Europe was conducted starting from April 18, 2024 (i.e., date of literature search), using PubMed/Medline, EMBASE, and Google Scholar. Included were European studies reporting quantitative data on the incidence and/or prevalence of C3G. The search string used in PubMed/Medline was «(complement 3 glomerulopathy OR c3g OR c3gn OR c3 glomerulopathy OR c3 glomerulonephritis OR membranoproliferative glomerulonephritis OR mpgn OR mesangio-capillary glomerulonephritis) AND (italy OR germany OR uk OR united kingdom OR england OR france OR spain OR sweden OR denmark OR norway OR finland OR netherlands OR switzerland OR belgium OR austria OR ireland OR portugal) AND (epidemiol* OR prevalence OR incidence OR mortality)». The same combination of terms was used in EMBASE. Studies identified with this strategy were reviewed and selected by 2 independent reviewers. After the selection process, 5 studies were identified with original data or contributing original calculation of the incidence and/or prevalence of C3G in Europe (2, 6, 16–18). The search strategies on other areas of investigations on C3G, including several aspects such as clinical management, disease prognosis, and burden, were not systematic. We combined the terms “complement 3 glomerulopathy”, “c3g” and “c3 glomerulopathy” with, in turn, specific items for each research area, including “treatment”, “clinical manifestation”, “disease outcome”, “quality of life”, “unmet needs”, etc. The main results of the literature review were summarized in a presentation and discussed during the kick-off meeting of the Steering Committee (see below).

Five main areas of investigation were defined, and the questionnaire for the first Delphi round was built by including a few relevant items for each research area. The main areas were: 1) epidemiology of C3G; 2) main patient characteristics; 3) disease outcomes; 4) treatment of C3G; 5) Other mixed aspects, including unmet needs, quality of life, and patient journey. The questionnaires (both for the first and second Delphi rounds) were iteratively revised by all the members of the Steering Committee until a final version was defined. One member of the Steering Committee (C.G.) conducted a pilot phase to test the questionnaires before starting the voting phases.

### 2.2 Selection of Steering Committee and panellists

The study was directed and managed by a Steering Committee involving two Italian nephrologists with expertise in the management of C3G (L.B. and G.C.) and two experts of the Delphi method, i.e., a biostatistician/epidemiologist (C.G.) and a Delphi Master (P.M.). After a bibliographic review, a kick-off virtual meeting of the Steering Committee was held on September 3, 2024. The Steering Committee was responsible for drafting and approving the final version of the survey questionnaires to be used in the Delphi iterations, for defining the main criteria of the Delphi study (e.g., assessment of requirements for the participation of the panellists, definition of consensus, etc.), for the revision and discussion of study results of each Delphi round, and for drafting and finalizing the current manuscript. Steering Committee members did not take part in the voting rounds of the Delphi consensus.

The criteria for panellists eligibility in the study, after discussion within the Steering Committee, was defined as “having diagnosed, taken in charge or treated at least 5 patients with C3G during the previous 5 years”. The corresponding preliminary question to assess expert eligibility was included in the first Delphi round. Panellists were initially identified by the Steering Committee and by Novartis team because of their deep knowledge of the specific therapeutic area and of the Italian scientific community at a regional level, focusing on qualitative expertise rather than a predefined sample size, while taking care to include experts from different geographic regions. Since healthcare management in Italy is highly regionalized, it was important to include key experts from different geographic areas, in order to reflect a nationwide rather than a localized perspective in the final consensus. Twenty-three nephrologists with clinical experience in managing patients with glomerular diseases were initially invited to participate through a contact e-mail that introduced the Delphi scheme, sent on November 28, 2024. The invitation process to panellists was centralized and managed by the Steering Committee.

### 2.3 Assessing consensus

Aim of each subsequent Delphi round was to achieve an agreement between voters on each item proposed. Most questions included in the Delphi survey were multiple-choice ones, mixed with some open-ended questions, and some statements. The latter, which was most frequently used during the second iteration, provided the panellists for expressing their agreement with specific assertions, through a 4-item Likert scale, as follows: “fully disagree”, “partially disagree”, “partially agree”, “fully agree”. In the analysis of responses, “full” or “partial” agreement and, on the other side, “full” or “partial” disagreement were joined together, in order to examine the level of consensus among binary responses. In the first iteration, an open-ended field after each question/statement was included, asking voters to supply any potential comment and allowing them to provide explanations or propose new items. In the second Delphi iteration, before each question or statement, we reported the results obtained for the corresponding topic during the first iteration. These were generally presented to the panellists through synthetic graphs or Tables.

Consensus in the Delphi rounds was defined according to the type of item (e.g., question or statement, qualitative or quantitative response, etc.) by simple statistical means, including the calculation of percentage of agreement or the median value of the responses. Analyses of responses to the survey were conducted on aggregate in order to guarantee the anonymity of voters. No subgroup analysis according to the characteristics of panellists (e.g., geographical area, type of hospital in which they worked, etc.) was conducted. The convergence criterium for consensus was pre-specified, i.e., defined according to the stability of results, to a threshold equal to 66.7% of the responders. Each questionnaire item was reclassified according to a Focus Level (19), as follows:

- Focus level A: corresponding to a high level of agreement between respondents (i.e., ≥66.7% of agreement on a specific response). Items in this level did not require any additional investigation in the subsequent iteration;
- Focus level B: corresponding to a moderate or high variability of replies between respondents (i.e., no single reply option reached the 66.7% threshold level of agreement). Items in this level required additional investigation in the subsequent iteration;
- Focus level C: corresponding to a high proportion (i.e., >33%) of non-responders or “I do not know” answers. Items in this level required additional investigation or rephrasing in the subsequent iteration.

Further, we defined “majority” an agreement ranging between 66.7% and 99.9% of the panellists and “full consensus” an agreement of all the panellists.

### 2.4 Participation

Participation was incentivized through a reimbursement fee from Novartis and by regular reminders sent to the panellists during the periods when the Delphi rounds were active, in order to promote responses.

After the contact e-mail explaining the project to the identified panellists, the second email included a link to the questionnaire of the first iteration of the Delphi study, that was active from November 29 to December 13, 2024. Out of 23 clinicians invited, 2 (8.7%) did not reply and 4 (17.4%) did not satisfy the inclusion criteria defined by the Steering Committee. Thus, the voting panel included 17 Italian experts. For the second Delphi round, an email with a link to the next questionnaire was sent on February 7, 2025, and the second iteration was active from this date to February 18, 2025. All 17 panellists participating to the first iteration completed the second Delphi round.

The Delphi study was based on the use of SurveyMonkey^®^, an *ad hoc* web platform where participants were able to complete the survey rounds.

### 2.5 Ethics

This work was conducted in accordance with the Declaration of Helsinki. The study is based on a survey of clinicians who contributed with their expertise and beliefs. No patients were involved in the Delphi process. The study was not submitted to an ethics committee nor to an institutional review board as this was deemed unnecessary on the basis of national regulation (i.e., Determina AIFA, 8^th^ August 2024, https://www.gazzettaufficiale.it/eli/id/2024/08/20/24A04320/sg; and Determina AIFA, 20^th^ March 2008, https://www.gazzettaufficiale.it/eli/id/2008/03/31/08A02109/sg). All panellists were informed by electronic means of the aims and funding of the research before initiating the survey, both in a contact e-mail and in an introductory page of the survey. Informed consent was collected from all participants through a specific question in an introductory page of the survey.

## 3 Results

The main characteristics of the panel are hereby described, according to the replies of participants to a few specific questions. Out of 17 clinicians, 4 worked in Northern Italy, 6 in Central Italy, 4 in Southern Italy, and 3 in the Isles (Sicily and Sardinia). All of them worked in reference centers accredited for rare diseases: 3 in a Scientific Institute/Hospital of Medical Research (Istituto di Ricovero e Cura a Carattere Scientifico, IRCCS), 9 in a university hospital, 4 in a general hospital and 1 in other settings. Patients with C3G managed and/or treated in the Department of each panellist in the year before the Delphi study were, on average, 5.0 (standard deviation, SD: 3.2), with a range of 1 to 10 patients.

The main findings of the study are described in detail below, separately for the 5 main areas of investigation.

### 3.1 Epidemiology of C3G

**Table 1** shows the consensus results on the epidemiology of C3G. Three items of the Delphi were focused on the incidence, prevalence and distribution of subtypes of C3G in Italy. During the first iteration, an agreement was achieved by the majority of panellists (14 out of 17, 82%) on an estimated incidence of C3G ranging between 1 and 1.5 new cases per million person-years, corresponding to 60–90 incident patient per year in Italy. Also, concerning the subtypes of C3G, most experts (94%) agreed during the first round that approximately three-fourths of cases present with C3GN and one-fourth with DDD. Most panellists indicated, during the first iteration, prevalence estimates below 5000 cases in Italy, but consensus was not reached. During the second iteration, the majority of experts (82%) agreed on an estimated number of about 2700 prevalent cases of C3G in Italy.

**Table 1:** Consensus findings on the epidemiology and main characteristics of patients with C3G.

Main area ❖ Sub-item	Reply achieving consensus ^a^	Round of consensus	% agreed ^b^
Epidemiology of C3G			
❖ A reliable estimate of C3G incidence is 1-1.5 new cases per million person-years, corresponding to about 60–90 new cases per year in Italy.	–	1	82%
❖ In Italy, an estimated number of around 2700 subjects have prevalent C3G	–	2	82%
❖ A plausible distribution of cases according to different C3G subtypes is about 75% C3GN and 25% DDD.	–	1	94%
Main patient characteristics			
❖ In which age group is C3G most frequently diagnosed?	In young adults (18–34 years)	2	94%
❖ At diagnosis, the sex distribution of C3G patients is quite balanced with, if any, a slight male majority	–	2	94%
❖ At diagnosis, C3G patients typically present with proteinuria and hematuria	–	1	100%
❖ At diagnosis, which proportion of patients present without proteinuria nor hematuria?	≤10% of patients	2	76%

C3GN, C3 glomerulonephritis; DDD, dense deposit disease.

^a^ The main reply is reported for multiple choice or open-ended questions, while statements did not require any reply.

^b^ All 17 panellists replied to all the items, and consensus was achieved for all the items reported in the Table.

### 3.2 Main patient characteristics

A few questions were aimed to define the main characteristics of patients at the diagnosis of C3G (**Table 1**). All but one panellists (94%) agreed that C3G diagnosis most frequently occurs in young adults (i.e., between age 18 and 34 years). A multiple-choice question was proposed during the first iteration on the sex distribution of patients at diagnosis, and the most frequent replies were joined in a statement (i.e., “At diagnosis, the distribution of C3G patients is quite balanced between males and females with, if any, a slight male majority”) that was approved by 94% of experts during the second round. There was full consensus that patients typically present with proteinuria and hematuria at C3G diagnosis, and the majority of panellists (76%) agreed that a small proportion of patients (i.e., ≤10%) have no such markers of glomerulonephritis at diagnosis.

### 3.3 Disease outcomes

**Table 2** summarizes the results on selected C3G outcomes. During the first round of the Delphi survey, three-fourths of panellists stated that, on the basis of their expertise and knowledge, 10-year overall survival is ≥85% in patients with C3G. An open-ended question was focused on the proportion of patients achieving 10-year renal survival, according to the definition used by Wong et al. (20). The mean and median proportions reported by panellists during the first round were 54% and 58%, respectively. Therefore, during the second round, we proposed the following statement: “About half of C3G patients maintain at least some kidney function 10 years after diagnosis (i.e., renal survival)”, and 94% of participants agreed with it.

**Table 2:** Consensus findings on disease outcomes and treatment of C3G.

Main area ❖ Sub-item	No. of respondents	Reply achieving consensus ^a^	Round of consensus	% agreed
Disease outcomes				
❖ Which proportion of C3G patients is alive 10 years after diagnosis (i.e., overall survival)?	16	≥85% of patients	1	75%
❖ About half of C3G patients maintain at least some kidney function 10 years after diagnosis (i.e., renal survival)	17	–	2	94%
❖ Is change in eGFR over time (slope) associated to the prognosis of C3G?	16	Yes	1	100%
❖ Is an improvement over time in proteinuria level favorably associated to the prognosis of C3G?	17	Yes	1	100%
❖ Which are the most important clinical outcomes to assess the efficacy/effectiveness of treatments in C3G patients?	17	1) Renal function/eGFR 2) Proteinuria	1	1) 100% 2) 88%
❖ Stabilization of eGFR over time is a key objective in the treatment of patients with C3G	17	–	1	100%
❖ Improvement in proteinuria level over time is a key objective in the treatment of patients with C3G	17	–	1	100%
Treatment of C3G				
❖ In consideration of your clinical expertise and knowledge, do you agree with the indications of the KDIGO Guidelines in first-line treatment of C3G?	17	Yes	1	94%
❖ How do you consider the efficacy/effectiveness profile of current treatments for C3G?	17	Unsatisfactory (very or partially)	1	71%

eGFR, estimated glomerular filtration rate.

^a^ The main reply is reported for multiple choice or open-ended questions, while statements did not require any reply.

Panellists expressed full consensus (100% agreement) during the first iteration that changes over time in eGFR and in proteinuria levels are both associated to the prognosis of C3G. In an open-ended question asking to report the most important clinical outcomes to assess treatment efficacy/effectiveness for C3G, eGFR/renal function (100% of panellists) and proteinuria (88%) were consistently mentioned. Further, in separate questions proposed during the first round, both the stabilization of eGFR and an improvement in proteinuria were considered key objectives of the treatment of C3G by the totality of experts (full consensus).

### 3.4 Treatment of C3G

In the first question of the area focused on treatment (shown in **Table 2**), we reported the key indications of the Kidney Disease: Improving Global Outcomes (KDIGO) Guidelines for the management of glomerular diseases (21). Almost all panellists (94%) supported the KDIGO indications. We explored the satisfaction of experts toward the efficacy/effectiveness and safety profiles of current treatments for C3G. The majority of panellists (71%) evaluated the efficacy/effectiveness of available treatments as totally (6%) or partially (65%) unsatisfactory during the first iteration. No agreement, on the other hand, could be reached over two rounds on the satisfaction toward the safety of current treatments, notwithstanding some re-wording of the corresponding item. Results were consistent between the two rounds, with 53% of experts in the first and 59% in the second iteration considering the safety of current treatments as partially or totally satisfactory. Thus, the item was not investigated further.

### 3.5 Other mixed aspects, including unmet needs, QoL, and patient journey

Two separate multiple-choice questions were posed in the first round to collect information on the key unmet needs related to C3G, i.e., separating clinical and patient needs (**Table 3**). Since replies were similar, indicating for both questions a need of effective treatments and of a prompt diagnosis, in the second round we joined the two items in a single statement (“Availability of specific and effective treatments, and a rapid and correct disease diagnosis, are the most important unmet needs related to C3G, both from a clinical and patient perspective”) that achieved full consensus (100%) of panellists. The majority of participants (94%) agreed that C3G impact on the quality of life of the patient by affecting, in particular, their psychological/emotional and physical spheres. Occupied patients with C3G may lose on average 1–5 working days per month due to disease-related factors (82% agreement), mainly because of visits and clinical/laboratory exams associated to C3G (88% agreement). Fourteen out of 17 experts (82%) agreed that a C3G Registry would be useful in Italy. Most of them (13/14, 93%) would prefer a national rather than a local/regional Registry, and consider a national Registry feasible (12/14, 86%). With reference to the patient journey, nephrologists (reported by 88% of experts) and family pediatricians (71%) were consistently reported as those who should ideally be involved in the first contact with C3G patients. As expected, there was full agreement (100%) that nephrologists are those physicians ideally responsible for diagnosis and for setting up the treatment of C3G patients.

**Table 3:** Consensus findings on other mixed aspects, including unmet needs, QoL, and patient journey.

❖ Sub-item	No. of respondents	Reply achieving consensus ^a^	Round of consensus	% agreed
❖ Availability of specific and effective treatments, and a prompt and correct disease diagnosis are the most important unmet needs related to C3G, both from a clinical and patient perspective	17	–	2	100%
❖ In occupied patients with C3G, how many working days are lost each month on average due to disease-related factors?	17	1–5 days	1	82%
❖ Which are the main disease-related factors responsible for the working days lost?	17	Visits and exams	1	88%
❖ The areas of quality of life that are most impacted by C3G are the psychologic/emotional and physical ones	17	–	2	94%
❖ Do you consider that a Registry of C3G would be useful in Italy?	17	Yes	1	82%
❖ Which type of Registry would ideally be preferred?	14	National Registry	1	93%
❖ In your opinion, which type of Registry would concretely be feasible?	14	National Registry	1	86%
❖ Which physician(s) should ideally be involved in the first contact with C3G patients?	17	1) Nephrologist 2) Pediatrician	1	1) 88% 2) 71%
❖ Which physician(s) should ideally diagnose C3G?	17	Nephrologist	1	100%
❖ Which physician(s) should ideally set up the treatment for C3G?	17	Nephrologist	1	100%

^a^ The main reply is reported for multiple-choice or open-ended questions, while statements did not require any reply.

## 4 Discussion

We explored the opinions of experts on the epidemiology of C3G, a topic for which quantitative information is lacking in Italy. Providing indications on the issue is of particular importance considering that epidemiological estimates of C3G are affected by local biopsy and referral procedures (2), and thus data from other countries are not easily applicable to Italy. A prompt agreement was reached on an incidence between 1 and 1.5 new cases per million person-years, in line with the (still limited) international literature (2, 6). This, combined to population data derived from the National Institute of Statistics (ISTAT, http://dati.istat.it/, last accessed on 17 March 2025), led to an estimated number of 60 to 90 new C3G diagnoses in year 2024 in Italy. Bibliographic data on C3G prevalence are scant and particularly heterogeneous across studies, ranging from 5 to 140 per million (2, 16, 22), again in the absence of specific information from Italy. After two Delphi rounds, over 80% of physicians agreed on an estimate of about 2700 alive patients with C3G in Italy. This is consistent with a simple computation combining broad estimates of 60 incident cases per year in Italy and a mean survival of 45 years after diagnosis. Uncertainties in defining the prevalence of C3G in Italy derive from the heterogeneity in the approach to renal biopsy and related analytic techniques, particularly immunofluorescence and electronic microscopy. While quantitative studies of higher evidence level are needed on the epidemiology of C3G, this consensus of clinical experts is useful to fix the complete gap of knowledge at a national level.

The study contributed additional information on the main characteristics of patients at C3G diagnosis. According to Italian expert nephrologists, this typically occurs in young adults, with a male-to-female ratio equal or slightly above unity. Approximately three out of four C3G cases are C3GN, and one out of four is DDD pattern, and only a minor proportion (i.e., ≤10%) of patients have no urinary abnormalities at diagnosis. For the latter subgroup, in open-ended questions on the diagnostic pathway, it emerged that referral to a nephrology specialist may occur due to a family history of disease, after suggestion by a general practitioner or family pediatrician. An Italian cohort study including 93 C3G cases (23), reported some major characteristics: 59% of patients were males; 73% had C3GN and 27% DDD; 91% had proteinuria; 87% had microhaematuria; age at diagnosis was not reported, but the mean age at disease onset was 18.2 years for C3GN and 15.3 years for DDD. Thus, our findings are consistent and further support previous indications on the baseline features of C3G patients in Italy.

C3G is a chronic disease with long-term survival and a generally delayed progression to end-stage kidney disease. Italian experts involved in this Delphi study agreed that 10-year overall survival exceeds 85% and renal survival is around 50%. This is largely consistent with the available literature (5, 6, 20, 22). There is thus the need of valid surrogate endpoints to evaluate in the short-term any response to treatment, particularly to facilitate the conduction of clinical trials (10). Many questions were submitted to Italian nephrologists on such issues, and, in most cases, a full consensus emerged between experts that both a stabilization in eGFR and an improvement in proteinuria values over time are favorable indicators of prognosis and treatment efficacy for C3G, as was previously shown for other chronic kidney diseases (8, 9). Further, all experts reported these endpoints as key targets of C3G therapies. The topic is relevant and very current, and our findings agreed with those from another recent panel (24). This notwithstanding, other related aspects are still undefined, for example the minimum/maximum clinically relevant cutoffs of variation in eGFR and proteinuria, and the issue requires further investigation.

A prompt and correct disease diagnosis and the availability of specific and effective treatments for C3G emerged as key disease-related unmet needs, with full consensus of experts. This is not surprising given the recognized challenges in C3G diagnosis (25), and the lack of approved therapies for C3G and of high-quality data (i.e., randomized clinical trials) at the time of the conduction of the Delphi study to guide disease management (21). An overall unsatisfaction toward traditional treatments was also confirmed by the replies on the corresponding efficacy/effectiveness, that was judged negatively by the majority of experts, whereas no consensus was reached on the overall safety profile of therapies available at study conduction. However, the therapeutic landscape of C3G has recently experienced a major shift with the approval and availability in Italy of iptacopan, a novel, first-in-class oral complement factor B inhibitor (26). Other relevant aspects achieving consensus included the impact of C3G on the occupational burden of patients (who are frequently diagnosed at an early working age, being otherwise fit), mainly due to the need of frequent visits and exams; the effect on quality of life of C3G, impacting both the emotional and physical spheres of the patients; and the anticipated usefulness of a national disease Registry. With reference to the latter aspect, we acknowledge the existence of the Italian Registry of MPGN (https://www.marionegri.it/registro-gnmp, last accessed on 20 March 2025), involving a network of about 15 Nephrology Units throughout the Country, and of the Italian Registry of Renal Biopsies, managed by the Italian Society of Nephrology. The latter is active since 2015 and has been recently made accessible to all the members. This may help to improve its coverage, potentially making the Registry an important resource to improve our knowledge of C3G, besides other kidney diseases, in Italy.

Our study has typical limitations of consensus methods (13). First, the investigation is based on opinions of C3G experts rather than on clinical/patient quantitative data. Therefore, its level of evidence is low. Second, Delphi studies may be prone to the risk of “forcing” consensus, particularly when influent panellists are participating. However, an anonymized eDelphi approach was used in our investigation, thus minimizing such risk. Third, the generalizability of this Delphi study outside of Italy is questionable, since the analysis involved only national nephrologists and, moreover, some aspects investigated were peculiar to Italy. On the other hand, potential strengths include the participation of a homogeneous panel of nephrologists satisfying *a priori* defined expertise criteria, the substantial participation (100% of first round panellists completed the second round, too) and high response rates, and the adherence to key requirements for Delphi study and to the ACCORD guidelines (12).

## 5 Conclusions

This Delphi analysis highlighted a strong agreement of nephrology specialists on several aspects of the epidemiology, management, and burden of C3G. In the current scarcity of quantitative, high-quality data, we provided a valuable basis—though based only on expert opinions—to improve disease knowledge. In particular, our study underlined the need of new effective therapies, specifically approved for C3G, at the time of study conduction, and consolidated the role of eGFR and proteinuria variations over time as surrogate disease outcomes. A paucity of Italian studies on C3G emerged in our bibliographic review preparatory to the Delphi survey (23, 27, 28). Therefore, our investigation may also be useful to stimulate discussion between national nephrologist, promote new studies on C3G and ultimately to enhance Italian research on this ultra-rare disease.

## Data Availability

The raw data supporting the conclusions of this article will be made available by the authors, without undue reservation.
